# Reactive Magnetron-Sputtered Tantalum–Copper Nitride Coatings: Structure, Electrical Anisotropy, and Antibacterial Behavior

**DOI:** 10.3390/nano15231813

**Published:** 2025-11-30

**Authors:** Paweł Żukowski, Vitalii Bondariev, Anatoliy I. Kupchishin, Marat N. Niyazov, Kairat B. Tlebaev, Yaroslav Bobitski, Joanna Kisała, Joanna Wojtas, Anna Żaczek, Štefan Hardoň, Alexander D. Pogrebnjak

**Affiliations:** 1 Lublin University of Technology, 38d Nadbystrzycka St., 20-618 Lublin, Poland; p.zhukowski@pollub.pl; 2 Department of Electrical Devices and High Voltage Technology, Lublin University of Technology, 38d Nadbystrzycka St., 20-618 Lublin, Poland; 3 Physico-Technological Center, Abai Kazakh National Pedagogical University, Dostyk, 13, Almaty 050010, Kazakhstan; ankupchishin@mail.ru (A.I.K.); marat--90@mail.ru (M.N.N.); tlebaev@mail.ru (K.B.T.); a.d.pogrebnjak@gmail.com (A.D.P.); 4 Faculty of Exact and Technical Sciences, University of Rzeszow, Pigonia 1 Str., 35-310 Rzeszow, Poland; ybobytskyy@ur.edu.pl (Y.B.); jkisala@ur.edu.pl (J.K.); 5 Faculty of Medicine, Collegium Medicum, University of Rzeszow, Kopisto 2a Ave., 35-315 Rzeszow, Poland; jowojtas@ur.edu.pl (J.W.); azaczek@ur.edu.pl (A.Ż.); 6 Department of Physics, Faculty of Electrical Engineering and Information Technology, University of Zilina, 010 26 Zilina, Slovakia; stefan.hardon@uniza.sk; 7 Institute of Materials, Faculty of Material Science and Technology in Trnava, Slovak University of Technology in Bratislava, 917 24 Trnava, Slovakia

**Keywords:** multilayer films, conductivity, photocatalytic activity, antibacterial activity, deposition

## Abstract

Tantalum nitride (TaN) coatings are valued for their hardness, chemical inertness, and biocompatibility; however, they lack intrinsic antibacterial properties, which limits their application in biomedical environments. Introducing copper (Cu) into the TaN matrix offers a potential solution by combining TaN’s mechanical and chemical durability with Cu’s well-documented antimicrobial action. This study explores how varying copper incorporation affects the structural, electrical, photocatalytic, and antibacterial characteristics of TaCuN multilayer films synthesized via reactive magnetron sputtering. Three thin TaCuN films were fabricated using a high-power reactive magnetron co-sputtering system, varying the Cu target power to control the composition. Structural and morphological analysis was performed using X-ray diffraction (XRD), scanning/transmission electron microscopy (STEM/TEM), and energy-dispersive X-ray spectroscopy (EDS). Electrical conductivity was studied along and across the film surfaces at temperatures ranging from 20 to 375 K using AC impedance spectroscopy. Optical and photocatalytic properties were assessed using UV–Vis spectroscopy and methylene blue degradation tests. Antibacterial activity against Staphylococcus aureus was analyzed under visible light using CFU reduction tests. XRD and TEM analyses revealed a multilayered four-zone architecture with alternating Ta-, Cu-, and N-rich phases and a dominant cubic δ-TaN pattern. The layers exhibited pronounced conductivity anisotropy, with in-plane conductivity (~10^3^ Ω^−1^ cm^−1^) exceeding cross-plane conductivity by ~10^7^ times, attributed to the formation of a metallic conduction channel in the mid-layer. Optical spectra indicated limited light absorption above 300 nm and negligible photocatalytic activity. Increasing the Cu content substantially enhanced antibacterial efficiency, with the highest-Cu sample achieving 95.6 % bacterial growth reduction. Morphological evaluation indicated that smooth film surfaces (Ra < 0.2 μm) effectively minimized bacterial adhesion. Reactive magnetron sputtering enables the precise engineering of TaCuN multilayers, combining high electrical anisotropy with robust antibacterial functionality. The optimized TaCuN coating offers promising potential in biomedical and protective applications where both conductivity and microbial resistance are required.

## 1. Introduction

The development of advanced biomaterials with intrinsic antibacterial properties has become an important area in the field of biomedical engineering and its relationship with the processes occurring on the surface of biocompatible materials, coatings, and films [1,2,3]. In particular, much attention is paid to the development of new versions of multifunctional thin-film coatings that not only provide mechanical strength and corrosion resistance but also suppress microbial colonization [4,5,6,7]. Among such materials, transition metal nitrides attract special attention due to their exceptional physical, chemical, and biological properties [8]. One of the representatives of nitrides, tantalum nitride (TaN), stands out for its high hardness, chemical inertness, and exceptional biocompatibility [9,10]. However, it has a small drawback, the lack of antibacterial properties, which limits its effectiveness in clinical settings where bacterial adhesion and biofilm formation prevail. One approach to addressing this issue is the incorporation of bactericidal metallic elements, such as copper (Cu), into a nitride matrix to produce novel antibacterial surfaces [11]. This approach has led to the development of tantalum copper nitride (TaCuN) coatings—materials that combine the structural advantages of TaN with the well-known antimicrobial properties of Cu [12,13].

Copper particles or their incorporation as implanted ions, or doping, during the preparation process are widely known for their ability to inactivate a wide range of pathogenic bacteria, including *Escherichia coli*, *Staphylococcus aureus*, *Salmonella enterica*, and *Pseudomonas aeruginosa* [14,15]. Its antibacterial efficacy is primarily due to mechanisms involving the disruption of bacterial membranes, the induction of oxidative stress through reactive oxygen species (ROS), and interference with critical cellular processes such as protein synthesis and DNA replication [16]. Unlike antibiotics, copper has unique mechanisms of action that make it less susceptible to bacterial resistance—a critical advantage in the modern era of growing antibiotic resistance [17]. Incorporating (doping) copper into a layer enables the creation of coatings that not only controllably release antibacterial ions but also maintain the mechanical integrity and durability required for medical applications [18].

TaCuN coatings are typically synthesized using reactive magnetron sputtering, allowing controlled adjustment of Cu [19]. This method allows researchers to adjust copper content and nitrogen stoichiometry to optimize antibacterial properties without compromising adhesion, hardness, or corrosion resistance. Studies have shown that higher Cu contents generally enhance antibacterial activity [20,21,22].

Despite some positive results and an understanding of the relationship between microstructure and the properties that determine the antibacterial properties of TaCuN coatings, key issues remain unclear, including the optimal copper concentration, the role of film crystallinity and surface roughness, the kinetics of Cu ion release, and the persistence of the antibacterial effect over time. Furthermore, the mechanisms by which TaCuN surfaces resist bacterial biofilm formation—one of the primary mechanisms of infection persistence—are still not fully understood. These identified challenges, therefore, highlight the need for additional research combining material characterization with microbiological analysis, photocatalysis, and optical properties to develop robust principles for the development of next-generation coatings.

Along with the improvements in mechanical, antibacterial, and photocatalytic properties, the different phase compositions and element ratios in the films can also lead to variations in conductivity. Since nitrides and metallic elements (Ta, Cu) each contribute differently to the electrical behavior, examining the conductivity of these complex TaCuN coatings is particularly important.

Copper-modified tantalum nitride coatings have recently gained attention due to their promising mechanical performance and emerging functional properties, including antibacterial activity and light-induced surface reactivity. Previous studies have shown that introducing Cu into TaN can modify the microstructure, affect crystallinity, and enhance biological activity [6], although the underlying mechanisms remain insufficiently understood. In particular, the interplay between phase composition, Cu distribution, surface morphology, and functional responses—such as conductivity, antibacterial effects, and photocatalytic behavior—has not been comprehensively investigated.

Despite several reports describing TaN- [9] or TaCu-based [6,23] coatings, systematic studies linking microstructure, chemical architecture, and functional performance within a single, well-controlled material system are limited. Furthermore, the influence of increasing Cu content on the optical/electronic properties and the antibacterial behavior of TaCuN thin films remains unclear.

This study addresses these gaps by examining TaCuN coatings with different Cu contents and analyzing how structural, compositional, and surface characteristics affect their mechanical, antibacterial, and photocatalytic properties. By correlating these features with the electrical behavior of the films, this study provides new insight into the multifunctional potential of TaCuN coatings. TaCuN coatings were intentionally deposited as multilayers. Multilayer architectures are known to improve mechanical stability, suppress crack propagation, and enable controlled spatial distribution of metallic and nitride phases. For TaCuN systems, alternating regions with different Ta, Cu, and N contents can also influence electrical behavior, ion mobility, and antibacterial activity by creating compositionally distinct functional zones. Therefore, a multilayer design was selected to better tune microstructure–property relationships within the films. To further adjust phase composition and stabilize the microstructure, the coatings were subjected to post-deposition annealing. Thermal treatment promotes grain growth, elemental redistribution, and the development of conductive channels within the layers. These changes can strongly affect both the electrical anisotropy and the antibacterial performance of TaCuN coatings. Photocatalytic or photoinduced charge-transfer processes also play an important role in antibacterial mechanisms. Under visible-light irradiation, conductive nitrides can accelerate photoactivated ROS formation, enhancing bacterial inactivation. Understanding the optical response and light absorption behavior of TaCuN coatings is, therefore, essential for evaluating whether any photo-assisted antibacterial effects can be expected under clinical lighting conditions.

## 2. Materials and Methods

### 2.1. Preparation of TaCuN-Based Multilayer Films

**Figure 1:**
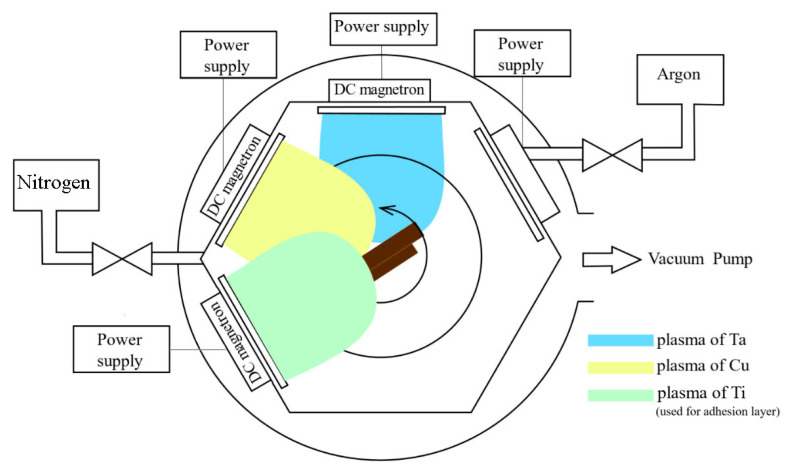
Schematic view of the multi-magnetron sputtering system.

The schematic representation of the experimental setup used for thin film deposition and subsequent annealing is shown in Figure 1. TaCu thin films were fabricated using the EPOS-PVD-440 system (Beams&Plasmas, Sankt Petersburg, Russia), which features three direct current (DC) magnetrons, each measuring 472 mm × 132 mm × 18 mm. The films were deposited on (100) silicon substrates pre-coated with a 100 nm titanium layer. Co-sputtering was performed using high-purity copper and tantalum targets (99.999%, Ulba LLP) to form the TaCuN monolayer. The deposition parameters are given in Table 1. The deposition was conducted over 180 min, under a base vacuum of 2 × 10^−5^ mbar and a working pressure of 1.7 × 10^−3^ mbar. During the process, the substrates rotated at a constant speed of 8 rpm, with the distance between the magnetrons and the substrate maintained at ~300 mm. Post-deposition, the films underwent annealing at 400 °C, 500 °C, and 600 °C for 1 h in a vacuum (2 × 10^−5^ mbar). These annealed samples were denoted by the format TaCuN-a, TaCuN-b, and TaCuN-c, respectively.

Post-deposition annealing was applied to promote phase rearrangement, relieve in-ternal stresses, and enhance the segregation or crystallization of Ta-, Cu-, and N-rich regions. Annealing is also known to influence conductivity pathways and Cu-related antibacterial activity.

### 2.2. Analysis of TaCuN Films Physico-Chemical Properties

The morphology and thickness of the TaCuN films were analyzed using a scanning electron microscope, specifically the JEOL JSM 7600F model (Tokyo, Japan). A metallographic process was employed to prepare the samples for cross-sectional viewing. This process involved grinding the samples with SiC abrasive papers, down to 1500 grit, followed by polishing with diamond colloidal suspensions. An energy dispersive spectroscopy system with an X-max 50 mm^2^ detector evaluated elemental composition and mapping. The constituent elements were quantified by averaging the measurements from five different regions.

The crystal structure of the coatings deposited was analyzed using X-ray diffraction with a Panalytical Empyrean X-ray diffractometer (Almelo, The Netherlands). The XRD analysis was conducted in a θ–2θ configuration with Cu K_α_ radiation (*λ* = 0.1541 nm). The X-ray diffraction analysis was performed with the X-ray source operating at 40 kV and 35 mA. The θ–2θ scan range was set between 20° and 100°, with a scan rate of 2°/min and a step size of 0.015°.

The microstructure of the films before and after ion implantation was characterized using a JEOL JEM-F200 (Hilmer)(Sydney, Australia) scanning/transmission electron microscope (S/TEM) operated at 200 kV. Samples were prepared for TEM using a FEI Helios G4 PFIB this uses a Xe plasma to mill the TEM lamella. The lamellae were thinned further in a Fischione 1040 Nanomill (Hanau, Germany) which uses a 500 eV Ar− ion beam to clean the surface of the sample. This JEOL F200 microscope used for imaging has a lattice resolution of 0.16 nm in ADF STEM mode and 0.1 nm in TEM mode at 200 kV. Determination of the amorphized areas in the layers was performed using high-resolution TEM (HRTEM) and elemental analysis was performed using a JEOL 100 mm^2^ silicon drift energy dispersive X-ray spectrometer (EDX). All EDX data were filtered to remove background counts and bremsstrahlung radiation.

### 2.3. Electrical Properties Tests

**Figure 2:**
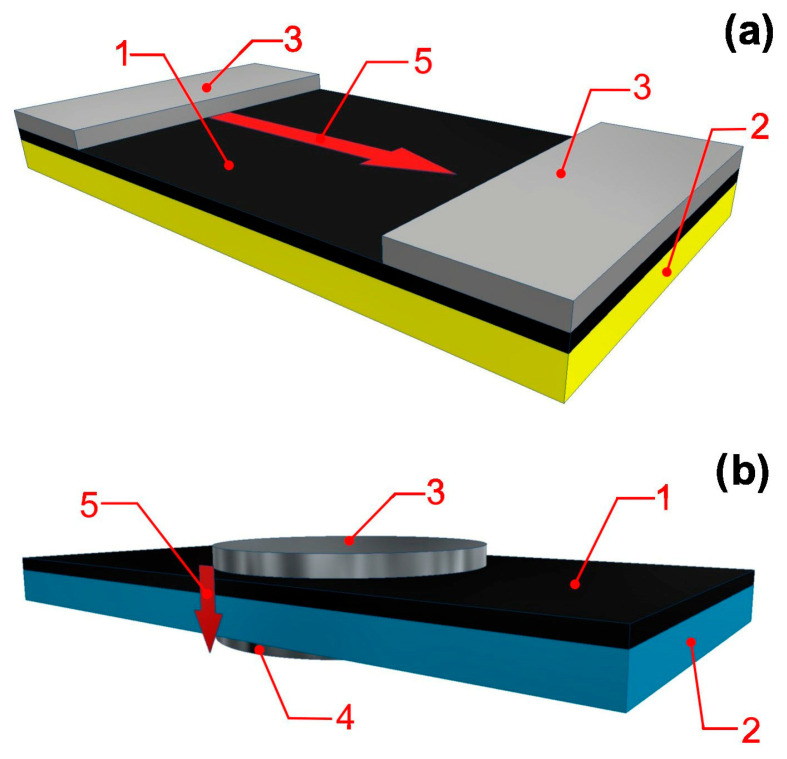
Views of the samples for measurements in two directions: along the layer on the dielectric substrate (**a**), and across the layer on the metallic substrate (**b**). 1. TaCuN layer with a thickness of approximately 750 nm, 2. substrate, 3. 4 contacts made of silver paste with a thickness of approximately 0.1 mm, 5. direction of current flow.

To investigate the potential occurrence of anisotropy in the electrical properties of TaCuN layers, two types of samples were prepared. For this purpose, two different types of substrates were placed side by side inside the chamber of the layer deposition system. One was made of glass, which is a dielectric material, and the other was a titanium (Ti) metal plate. The surfaces of both substrates were polished. TaCuN layers were deposited onto both substrates simultaneously in a single sputtering process. Electrical contacts were then applied to the deposited layers. Views of the samples with applied contacts are shown in Figure 2. From Figure 2a, it can be seen that the electrical properties of the layer deposited on the dielectric material were examined with current flowing parallel to the surface of the layer. The layer deposited on the metallic substrate was tested with current flowing perpendicular to its surface (Figure 2b).

**Figure 3:**
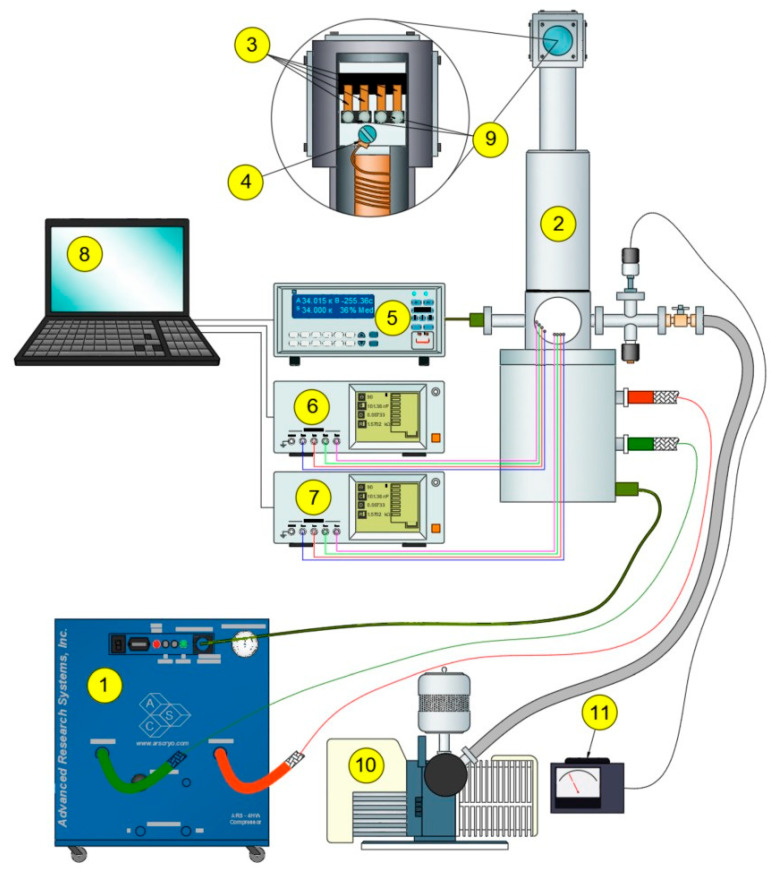
Schematic of the setup for studying the electrical properties of nanocomposites. 1. cryocooler, 2. measurement head, 3. measurement contacts, 4. temperature sensor, 5. temperature controller, 6. and 7. impedance analyzers, 8. computer, 9. tested samples, 10. vacuum pump, 11. vacuum gauge.

For the study of AC electrical properties, a measurement setup was used [24,25]; the schematic of which is shown in Figure 3.

The setup included a closed-cycle helium cryocooler CS 204AE-FMX-1AL from Advanced Research Systems, Inc., Macungie, PA, USA (1). The cryocooler is connected to the measurement head (2), which contains the contacts for layer measurements (3). A temperature sensor (4) is placed near the contacts and is connected to a temperature controller (5). The temperature range for measurements was from 20 K to 375 K. The temperature was changed during measurements with steps of 2 K in the 20–40 K range, 3 K in the 40–151 K range, and 7 K in the 151–375 K range. Electrical property measurements were performed using 3532 LCR HiTESTER impedance analyzers from Hioki (Ueda, Japan) (6,7). A computer (8) was used to control the measurement process and acquire the collected data.

### 2.4. Photocatalytic Tests of TaCuN Coatings

Photocatalytic properties were evaluated in the degradation reaction of methylene blue (MB). Samples of TaCuN (TaCuN-a, TaCuN-b, TaCuN-c) were placed in a quartz beaker, where MB solution was added (20 cm^3^, 5 × 10^−5^ mol dm^−3^, pH = 6). The MB solution without sample was irradiated parallelly to measure MB photolysis. The irradiation was performed with a handmade blue diode lamp as a light source (power density 8.78 W·m^−2^ measured by the Peak Tech (Ahrensburg, Germany) 5025 digital lux meter, wavelength 455 nm) held at 30 cm from the sample. The MB decay was monitored by spectrophotometric measurements (VWR (Radnor Township, PA, USA) UV-VIS 3100 PC spectrophotometer) at regular time intervals. External standards of five concentrations ranged from 5 × 10^−5^ to 5 × 10^−6^ mol·dm^−3^.

### 2.5. Antibacterial Activity of TaCuN Layers

For antimicrobial assays, Gram-positive strain *Staphylococcus aureus* (ATCC 25923) from the collection of the Department of Microbiology (University of Rzeszów, Rzeszów, Poland) was used. Nutrient broth and nutrient agar (BTL, Warsaw, Poland) or phosphate buffered saline (PBS, Sigma Aldrich, Milwaukee, WI, USA) were used for bacteria culturing and serial dilutions, respectively. All assays were carried out in a laminar flow hood (BioTectum, Bielsko-Biała, Poland). Before the experiment, the plates were sterilized by rinsing in 70% EtOH and UV irradiation for 20 min.

**Figure 4:**
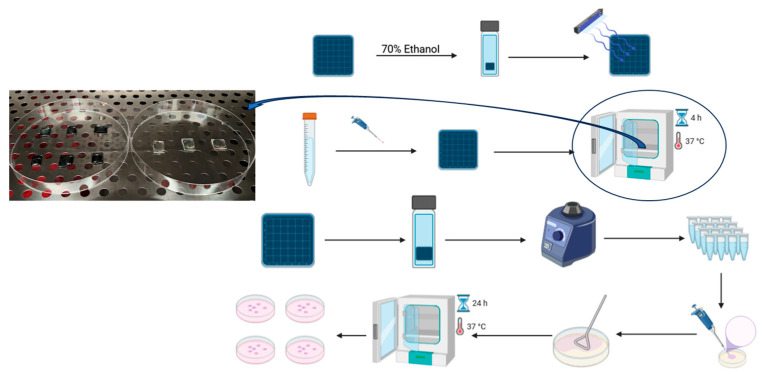
Schematic diagram of the experiment to assess antibacterial activity—Incubation of TaCuN-b, TaCuN-c, and K (control-glass) with culture of *S. aureus*.

The overnight culture of bacteria incubated in aerobic conditions at 37 °C with shaking, was diluted to give an initial concentration about 2.5–10 × 10^4^ cells/mL and poured onto the sterile coatings of test plates (TaCuN-b and TaCuN-c) and control plates (K, glass plate) in 3 replicates (Figure 4). The plates were incubated at 37 °C for 4 h with a relative humidity 85–90% (Thermo Fisher incubator, Waltham, MA, USA). During the incubations, samples were irradiated with a handmade blue diode lamp of 8.78 W m^−2^ power density (455 nm). After 4 h of incubation, each plate was placed in a Falcon tube containing 5 mL of PBS. The tubes were vortexed at 1400 rpm for 1 min to extract all bacteria. Serial ten-fold dilutions of bacteria were prepared and spread on the nutrient agar plates and incubated overnight at 37 °C for 24 h. The results were determined by counting the bacteria that grew on the plates as single colonies (colony forming units, CFU) (Figure 4). The percentage of growth reduction (%R) was calculated according to the formula:

(1) $$\%R=\frac{\left(CFU_{control}-CFU_{sample}\right)}{CFU_{control}}\times 100\%$$

## 3. Results and Discussion

### 3.1. Physico-Chemical Properties of TaCuN Films

**Figure 5:**
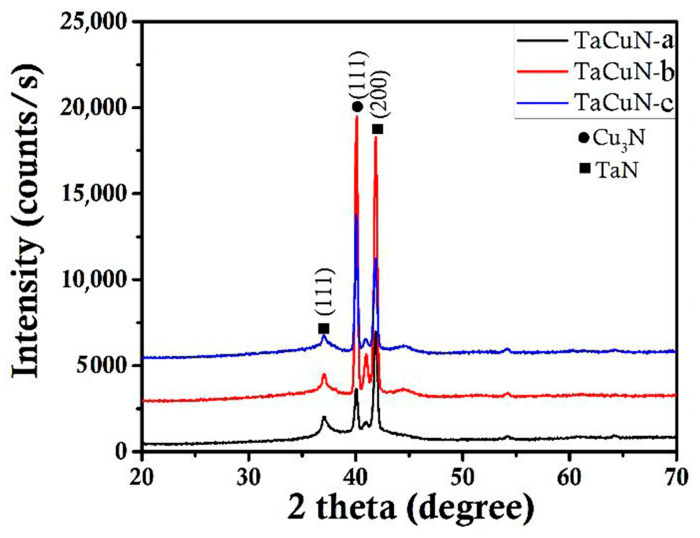
The XRD patterns of the TaCuN thin films.

The XRD patterns obtained for the TaCuN-a, TaCuN-b, and TaCuN-c coatings confirm the formation of a cubic δ-TaN structure, characterized mainly by the (111) and (200) diffraction peaks (Figure 5) [6,26]. Increasing the Cu content results in a clear improvement in crystallinity for the TaCuN-b sample, which is reflected by narrower and more intense diffraction peaks—especially the (200) reflection—indicating a stronger preferred orientation and enhanced grain growth [26]. In comparison, TaCuN-a exhibited broadened and weaker peaks typical of poorly crystalline material, whereas TaCuN-c showed a reduction in peak intensity and additional broadening, which may be attributed to lattice distortions or microstructural defects introduced at higher Cu concentrations. Since the peak positions remain nearly unchanged for all samples, it can be concluded that copper does not markedly modify the TaN lattice itself but influences the grain structure and orientation. The small peak detected at 2θ ≈ 40–41° corresponds to the cubic Cu_3_N phase, confirming the presence of Cu-enriched regions. Its intensity increases systematically with higher Cu sputtering power (0.26, 0.44, and 0.75 kW).

The texture evolution is further supported by the intensity ratios I(200)/I(111), which change from 4.3 (TaCuN-a) to 11.7 (TaCuN-b), and then decrease to 5.6 (TaCuN-c). This trend suggests that moderate Cu incorporation promotes (200)-oriented growth, while excessive Cu leads to structural disturbances that weaken the texture.

The lattice parameter calculated from the main diffraction peaks remains nearly unchanged across the coatings: for TaCuN-a and TaCuN-b, a = 4.255 Å, and for TaCuN-c a slight increase to 4.257 Å was observed. This small variation indicates that the TaN-based cubic phase is stable over the entire range of Cu deposition powers [27], and that Cu atoms do not significantly replace Ta in the lattice but instead tend to form Cu-rich secondary phases.

**Figure 6:**
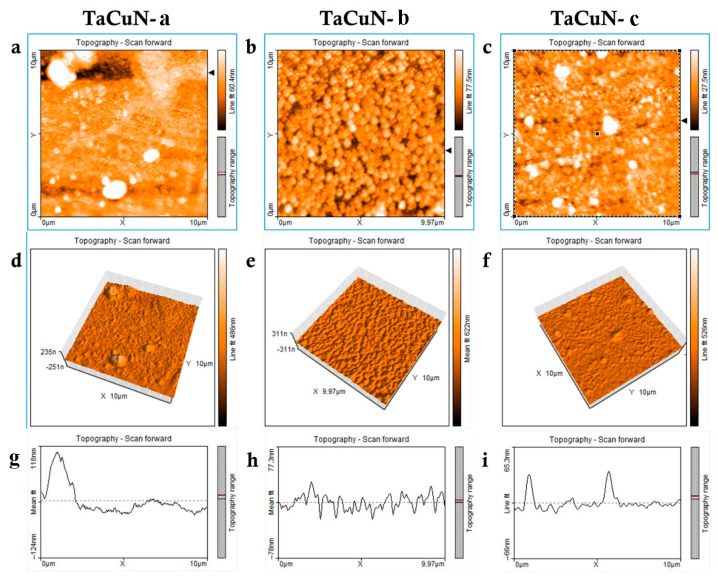
Geometric structure of the TaCuN coatings surface: (**a**–**c**) 2D images of field observations 10 μm × 10 μm; (**d**–**f**) 3D images; (**g**–**i**) Surface profile along the line of 2D images.

The AFM characterization of the TaCuN thin films (TaCuN-a, TaCuN-b, and TaCuN-c), presented in Figure 6, reveals clear differences in surface morphology and roughness that arise from varying copper content. The TaCuN-a coating exhibits a generally smooth surface interrupted by isolated large protrusions, likely associated with particle agglomeration or irregular nucleation events. TaCuN-b shows a more uniform and densely packed granular structure, indicating more stable surface growth. In contrast, TaCuN-c, which contains the highest amount of copper, presents a heterogeneous topography marked by numerous sharp peaks and depressions, suggesting phase separation or non-uniform grain coalescence [28].

Quantitative roughness metrics further highlight these distinctions. TaCuN-b shows the highest average roughness (*S*_a_ = 9.1 nm) and moderate peak height (*S*_p_ = 48.9 nm), reflecting a compact surface with consistent nanoscale features. TaCuN-a has a slightly lower *S*_a_ (7.8 nm) but exhibits the tallest surface peak (*S*_p_ = 107.1 nm) and the deepest valley (*S*_v_ = −44.4 nm), confirming the presence of occasional large irregularities. TaCuN-c demonstrates the lowest overall roughness values (*S*_a_ = 3.4 nm, *S*_q_ = 5.5 nm), indicating a generally smoother surface, although its peak height (*S*_p_ = 63.7 nm) still points to some localized unevenness. The *S*_q_ values follow the same trend, supporting the observation that TaCuN-a and TaCuN-b possess more pronounced surface texture compared to the relatively flat TaCuN-c. A full set of parameters is summarized in Table 2.

These morphological features have direct implications for the functional behavior of the coatings [6]. The balanced roughness and uniform structure of TaCuN-b may enhance adhesion, wear resistance, and overall functional stability, making it an attractive option for applications requiring reliable surface performance. Conversely, the more irregular surfaces of TaCuN-a and TaCuN-c, while potentially offering increased surface area beneficial for antibacterial action, may negatively affect mechanical integrity or optical uniformity. This underscores the importance of precise control of Cu incorporation during deposition to achieve an optimal compromise between surface structure and targeted properties of TaCuN coatings.

**Figure 7:**
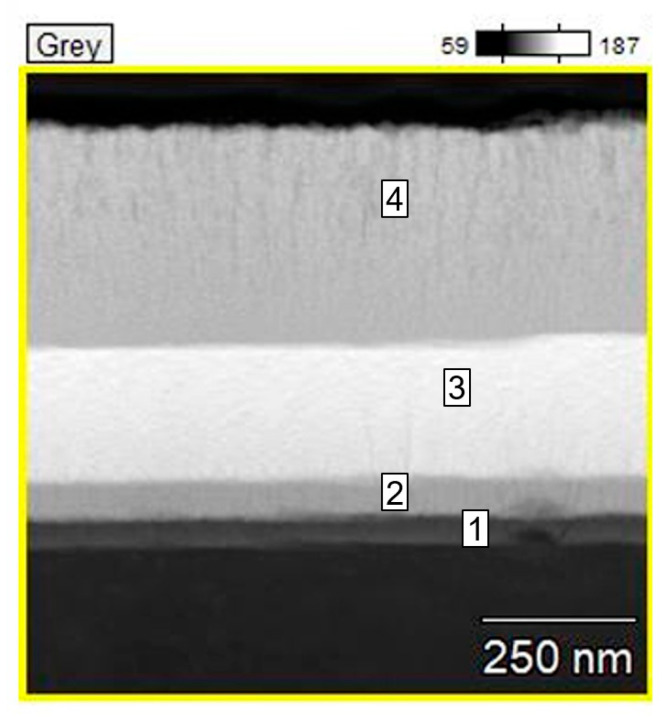
XEDS-derived elemental composition of the TaCuN-b thin film. The numbered regions indicate the individual areas whose chemical compositions are provided in Table 3.

The cross-sectional STEM image and XEDS elemental maps provide a clear view of the multilayered structure in the TaCuN-b thin film created by magnetron sputtering. As shown in Figure 7, the grayscale image reveals four distinct layers: a bright top layer, two middle layers with varying contrast, and a darker bottom layer near the substrate. These visual contrasts align well with the elemental differences identified through EDS analysis, indicating that the layers were deposited in a controlled and deliberate manner, resulting in a complex architecture.

Thus, the bottom layer (Layer 1), directly in contact with the substrate, is composed of titanium (77 at.%), while the detected nitrogen originally occurs by overlapping signals from the next layers. Layer 2 contains a balanced mix of tantalum (29 at.%) and copper (40 at.%), along with nitrogen (12 at.%), suggesting this is one of the main TaCuN functional layers with minor surface oxidation. A small amount of carbon (8 at.%) might belong to surface contamination. In Layer 3, tantalum becomes more prominent (42 at.%) while copper content slightly decreases (37 at.%), and nitrogen disappears—this may indicate a transition zone within the film. Finally, in Layer 4, copper again dominates (42 at.%) alongside nitrogen (12 at.%) and a reduced amount of tantalum (4 at.%), forming the outermost surface layer. The detailed elemental breakdown is presented in Table 3.

The EDS maps visually confirm these compositional patterns. Tantalum and copper are spread throughout the main film body, while nitrogen is concentrated in the outer layers (Layers 2 and 4), indicating successful reactive sputtering. Carbon is present in all layers but becomes most concentrated at the surface, likely from ambient exposure after deposition.

**Figure 8:**
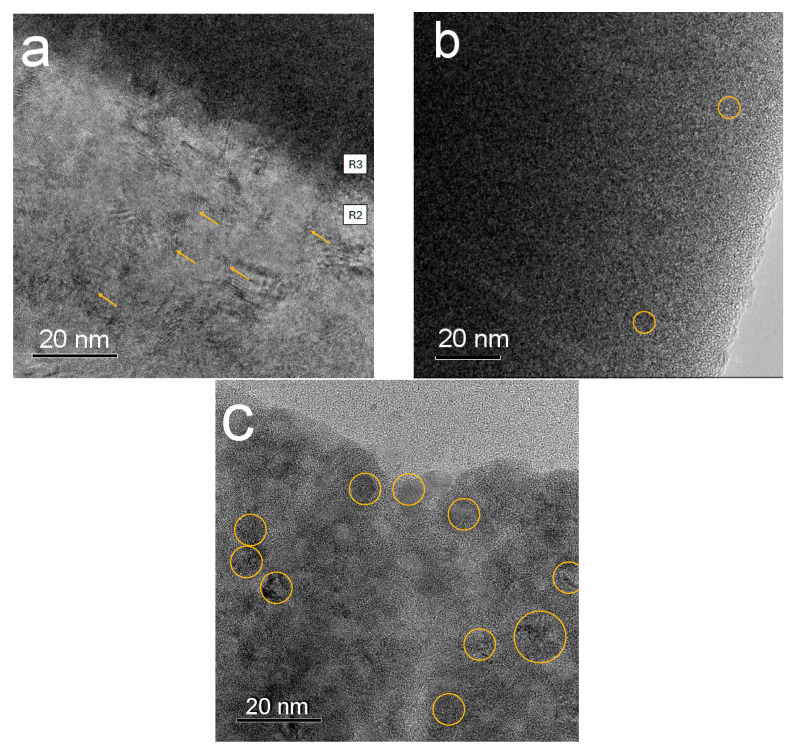
High-resolution transmission electron microscopy of the R2 region (**a**), R3 region (**b**), and top layer (R4 region) (**c**) of the TaCuN-b thin film.

In the top layer (Figure 8c), we see that the sample is composed of crystals ~ 10 nm in diameter, some are circled for clarity. It is important to note that the HRTEM also shows a large presence of amorphous material surrounding these crystallites.

**Figure 9:**
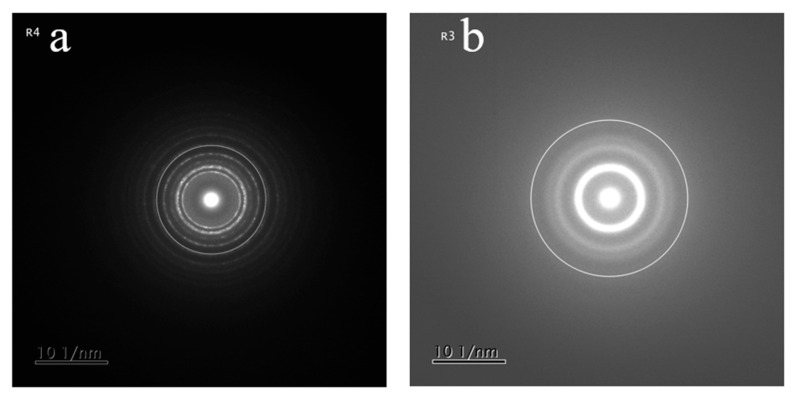
SAED analysis of multilayered TaCuN-b thin films (**a**) from the top layer showing polycrystalline rings and (**b**) from the third layer showing diffuse amorphous rings.

The diffraction pattern from the fourth layer (Figure 9a) indicates a nanocrystalline region that cannot be matched to cubic or hexagonal crystal structure whereas the diffraction pattern from the third layer (Figure 9b) shows broad diffuse rings consistent with amorphous material or material with no long-range order. High-resolution transmission electron microscopy (HRTEM) analysis of region R3 revealed a material with very low crystallinity, predominantly amorphous in nature, with localized areas exhibiting short-range order (indicated by circles) (Figure 8b). The microstructure of this region can, therefore, be classified as glassy. In contrast, the HRTEM image of region R2 (Figure 8a) showed a fully polycrystalline structure, with no amorphous phase detected. The microstructure consists of fine grains with an average size of approximately 15–20 nm, within which nanotwins were identified (arrowed).

The increase in the concentration of Cu and N, with an almost equal concentration of Ta, as can be seen from Table 4. This is also in comparison with the results of XRD analysis, which can be associated with the antibacterial ability of TaCuN films (moreover, both the concentration of copper nitride and the concentration of local copper areas increase, the peak from which we do not see in the XRD spectra due to the low copper concentration for the X-ray diffraction method). It can even be said that the contribution of Cu and N in the resulting film is very significant for the bacterial properties, as will be shown further.

### 3.2. Electrical Properties

The aim of this chapter was to investigate the potential occurrence of conductivity anisotropy in thin nanocrystalline layers. The possibility of anisotropy in such layers was suggested by the results of percolation phenomenon simulations presented in the publication [29]. In that publication, it was established that in composite or nanocomposite layers with large differences between their thickness and length, the percolation channel for current flow forms earlier in the transverse direction than along the layer. This means that above the percolation threshold, the conductivity in the transverse direction will be higher than along the layer. This is easy to understand, considering that a short percolation path will form more quickly than a long one.

**Figure 10:**
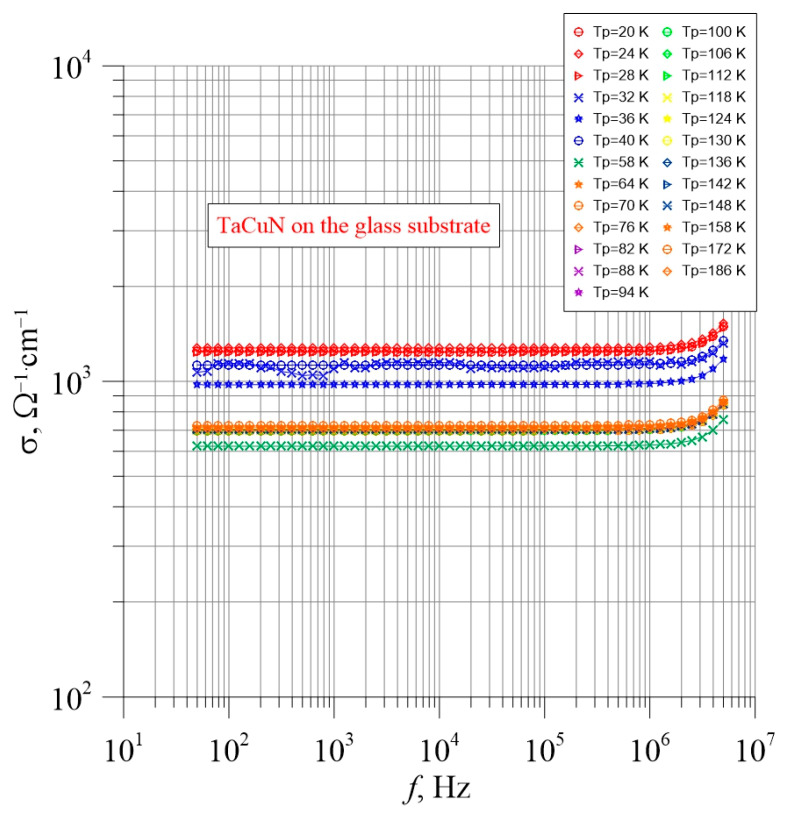
Layer deposited on the glass surface. Conductivity measurements along the layer.

Figure 10 shows the frequency–temperature dependencies of the layer deposited on the glass substrate. The measurements were performed along the surface of the layer.

**Figure 11:**
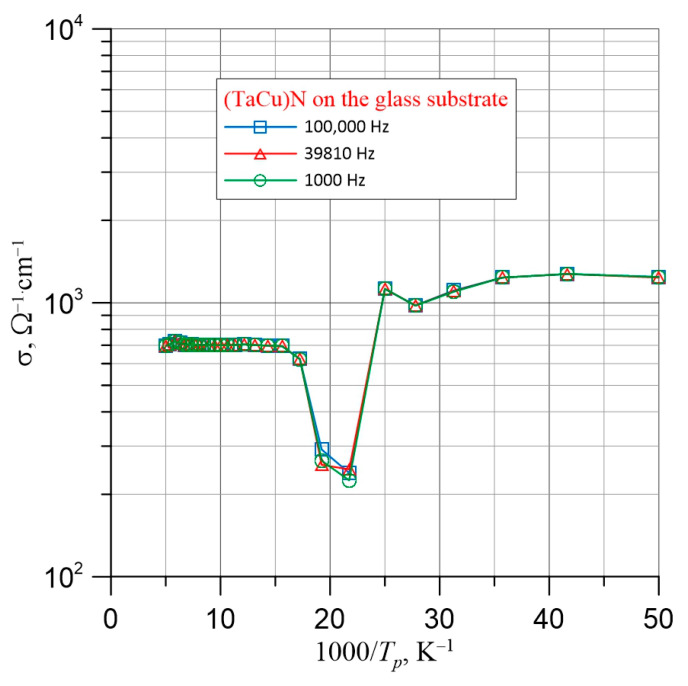
Arrhenius plot for conductivity measured along the layer. Measurement frequencies: 1000 Hz, 39,810 Hz, and 100,000 Hz.

From Figure 10, it can be observed that in the frequency range from 50 Hz to approximately 10^6^ Hz, the conductivity is independent of frequency. In the region above 10^6^ Hz, a slight increase in conductivity is observed. From Figure 10, it can be seen that an increase in temperature causes the conductivity to increase by approximately three times. This indicates dielectric-type conductivity. To determine the temperature dependence of conductivity, Arrhenius plots (Figure 11) were constructed for conductivities measured at frequencies of 1000 Hz, 39,810 Hz, and 100,000 Hz—that is, from the range where frequency has no significant effect on conductivity. As seen in Figure 11, the Arrhenius plots at all frequencies exhibited a similar shape.

From Figure 11, it can be seen that the dependence of conductivity on the inverse temperature (1000/*T*) is non-monotonic. A distinct minimum occurs around the value of 1000/*T* = 22. The presence of this minimum is likely related to the nanostructure of the layers, as observed in high-resolution transmission electron microscopy (HRTEM) images (Figure 8).

The distinct minimum is associated with carrier scattering on nanoclusters. This is a quantum phenomenon related to the wave nature of electrons. At low temperatures, the electron wavelengths are longer than the dimensions of the nanoparticles, resulting in weak scattering. As the temperature increases, the wavelength decreases, and scattering intensifies starting from around 1000/*T* ≈ 25, reaching a maximum at approximately 1000/*T* ≈ 21. At this point, the conductivity exhibits a pronounced minimum. Further temperature increase continues to reduce the wavelength, and scattering on the nanoinclusions gradually diminishes.

Scattering begins when the electron wavelengths become comparable to the dimensions of the nanoinclusions. Based on the temperature value at which the decrease in conductivity starts, visible in Figure 11—approximately 40 K—we can calculate the electron wavelength and, consequently, estimate the size of the nanoinclusions. As is well known, the wavelength of an electron accelerated by a potential difference *U* is given by:

(2) $$\lambda =\frac{h}{\sqrt{2m_{e}eU}}$$

where h—Planck’s constant, *m*_e_—electron mass, e—electron charge, and *U*—potential difference.

In Equation (2), e*U* = *E* is the energy gained by the electron from the electric field after passing through the potential difference *U*. In our case, this corresponds to the thermal motion energy, given by the formula:

(3) E=kT

where k—is the Boltzmann constant, and *T*—is the temperature. Substituting this value into Equation (2), we obtain:

(4) $$\lambda =\frac{h}{\sqrt{2m_{e}kT}}$$

Substituting the numerical values of the physical constants h, m_e_, k, and the temperature at which the decrease in conductivity begins (*T* = 40 K) into Equation (4), we calculate the electron wavelength as approximately 20 nm. This means that the nanoinclusions in the layer have dimensions of 20 nm or less. This is consistent with the results obtained using HRTEM and SAED, see Figure 8 and Figure 9.

**Figure 12:**
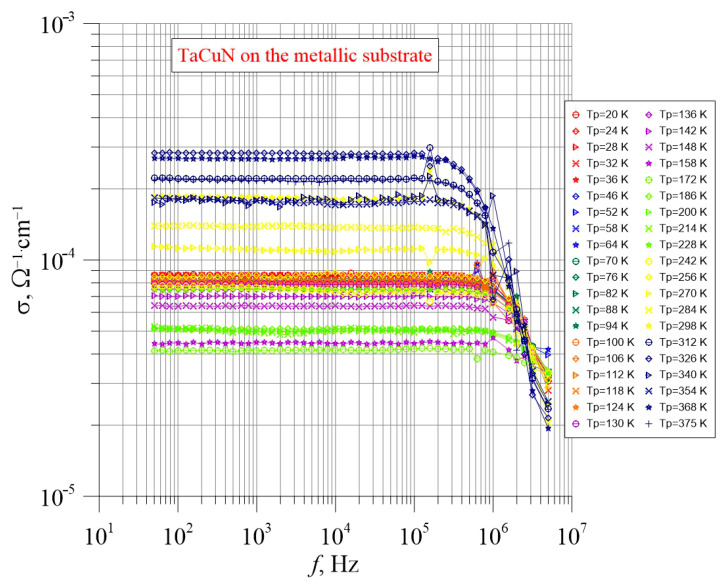
TaCuN layer deposited on a metallic substrate. Measurements across the layer.

Figure 12 shows the frequency–temperature dependencies of the layer deposited on the metallic substrate. Conductivity measurements were performed across the layer.

From Figure 12, it can be seen that in the frequency range up to approximately 2 × 10^5^ Hz, the conductivity in the transverse direction is independent of frequency. At higher frequencies, a slight decrease in conductivity is observed. Compared to the layer deposited on the dielectric substrate, the transition from constant conductivity to frequency-dependent behavior occurs at a somewhat lower frequency, around 2 × 10^5^ Hz. For the layer on the metallic substrate, a decrease in conductivity is observed, whereas the layer on the dielectric substrate exhibits an increase in conductivity.

The most significant observation is the change in conductivity values when transitioning from measurements along the layer to measurements across the layer. A comparison of the results presented in Figure 10 and Figure 12 shows that the conductivity of the layer deposited on glass is nearly 10^7^ times higher than that of the layer on the metallic substrate. The conductivities are approximately 10^3^ Ω^−1^cm^−1^ and 10^−4^ Ω^−1^cm^−1^, respectively. To analyze the origin of the observed large difference in conductivity between measurements along and across the layer, a cross-sectional image of the layer was taken using STEM.

Figure 7 shows a cross-section of the layer obtained using STEM. From the image, the thickness of the layer was determined to be (750 ± 50) nm. The image reveals that the layer is non-uniform and consists of four distinct regions. The chemical composition of each region, expressed in atomic percent, is given in Table 3.

The cross-section of the layer reveals four regions differing in chemical composition. According to Table 3, the first region, which is directly adjacent to the substrate, represents a transition zone between the substrate and the deposited layer. It contains approximately 77 at.% Ti, which is the material of the substrate. Additionally, it includes small amounts of Ta (8 at.%), N (8 at.%), and O (5 at.%). Oxygen is present in trace amounts across all regions, likely originating from residual vacuum gases. In the second region, the Ti content decreases to around 6 at.%, while the concentrations of the deposited metals Ta (29 at.%) and Cu (40 at.%) increase significantly. This region also contains N (12 at.%) and C (8 at.%). A similar composition is observed in the fourth region—Ta (29 at.%), Cu (42 at.%), N (12 at.%), and C (10 at.%). Based on their chemical composition, regions 2 and 4 consist of both metallic components and metal carbides and nitrides. Considering the typical stoichiometry of metal oxides, nitrides, and carbides present in these regions, it can be assumed that approximately 25% of the metal atoms in regions 2 and 4 are bound in compound form.

The remaining approximately 50% of metal atoms are not part of compounds. This means that these regions can be considered as metal–dielectric composites with a composition of (Ta, Cu)_0.5_(Ta, Cu, N, C, O)_0.5_. In terms of elemental composition, the third region stands out. The contents of carbon and oxygen are comparable to those in regions two and four. However, nitrogen is absent in this region, which could be due to nitrogen diffusion toward the layer boundaries. This suggests that region three, unlike regions two and four, does not contain metal nitrides. On the other hand, the carbide content remains nearly unchanged. The composition of region three can be represented as (Ta, Cu)_0.7_(Ta, Cu, N, C, O)_0.3_. A similar situation was observed in the publication [30,31]. Such a composition of the third region indicates a lower content of metal compounds and a higher content of metallic components compared to regions two and four. This results in an increase in conductivity relative to regions 2 and 4.

During the measurements along the surface of the layer, the equivalent circuit can be represented as a parallel connection of regions 2, 3, and 4. Two of them, regions 2 and 4, have lower conductances *G*, while region 3 has a higher one. The parallel connection of these three regions results in a total conductance *G* that is even greater than the conductance of the highly conductive region 3 alone.

**Figure 13:**
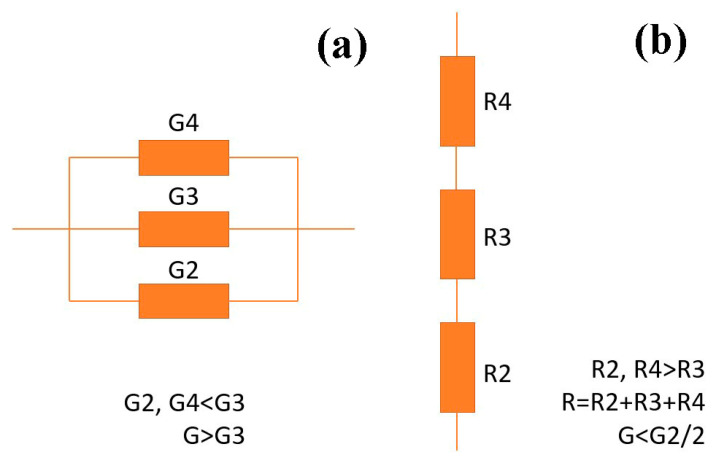
(**a**) Equivalent parallel circuit, measurements along the layer, measured on dielectric (glass) substrate. (**b**) Equivalent series circuit, measurements across the layers from surface to metal substrate.

Conduction takes place mainly through this region, which forms a conductive channel connecting the electrodes. The equivalent circuit of such a system is shown in Figure 13a.

The total conductance for measurements along the layer can be expressed as:

(5) $$G_{P}=G_{2}+G_{3}+G_{4}$$

where: *G*_2_, *G*_3_, *G*_4_—conductances of layers 2, 3, and 4. It should be noted that:

(6) $$G_{3}>>G_{2}+G_{4}$$

Hence:

(7) $$G_{P}\approx G_{3}$$

Measurements in the transverse direction, perpendicular to the layer surface, cause the current to flow through three layers connected in series. The equivalent circuit of such a system is shown in Figure 13b. In this configuration, the resistances of the individual components add up, resulting in the total resistance being the sum of the resistances of the three regions 2, 3, and 4.

(8) $$R_{S}=R_{2}+R_{3}+R_{4}$$

Since:

(9) $$R_{2}+R_{4}>>R_{3}$$

The total resistance in measurements perpendicular to the layer surface is given by:

(10) $$R_{S}≃R_{2}+R_{4}$$

The conductance in measurements perpendicular to the layer surface is:

(11) $$G_{S}≃\frac{1}{R_{2}+R_{4}}<<{\frac{1}{R}}_{3}$$

This means that the effective conductivities of the systems shown in Figure 13a,b will differ significantly. Specifically, the conductivity of the system during measurements along the layer surface (Figure 13a) will be higher than in the direction perpendicular to the layer surface (Figure 13b). From Figure 7, it can be seen that the thickness of area 3 is about 1/3 of the total layer thickness. Considering that the effective conductivity measured along the layer is about 10^7^ times greater than through the layers and is approximately 10^3^ Ω^−1^cm^−1^, it can be estimated that the conductivity of the highly conductive area (area 3 in Figure 7) is about 3·10^3^ Ω^−1^cm^−1^, while the conductivity of areas 2 and 4 from Figure 7 is about 5·10^−5^ Ω^−1^cm^−1^.

### 3.3. Optical and Photocatalytic Properties

**Figure 14:**
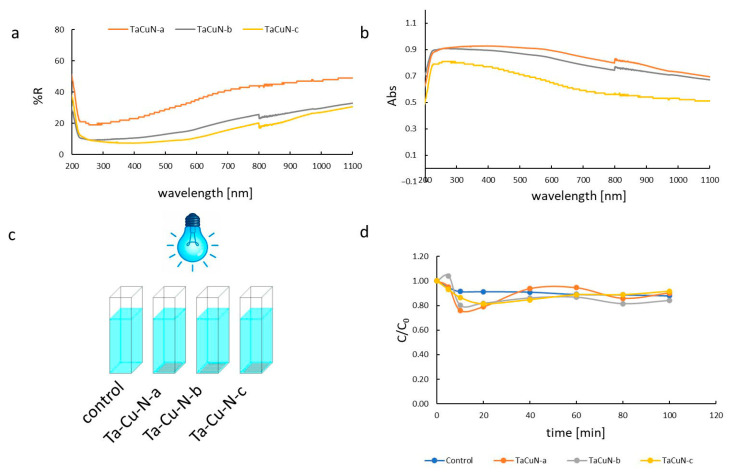
Layer deposited glass UV-Vis DRS spectra—reflectance (**a**), absorption (**b**); schematic presentation of photocatalytic experiment (**c**); Change in MB concentration in the presence of samples Control (glass, blue), TaCuN-a (orange), TaCuN-b (grey), TaCuN-c (yellow) under 455 nm irradiation (**d**).

Despite the high in-plane conductivity, photocatalytic activity was negligible. Optical reflectance data showed that the films exhibited high reflectance in the visible range, particularly above 300 nm (Figure 14a,b). Each sample showed a significant decrease in reflectance in the UV range (200–300 nm). Above 300 nm, the reflectance value gradually increased throughout the analyzed range. The maximum absorbance value was observed at a wavelength of 250 nm and then decreased systematically. Sample TaCuN-c is characterized by the highest absorbance of all samples.

The photocatalytic activity of surfaces was studied in the degradation reaction of MB aqueous solution (Figure 14c). The reaction was carried out under weak acidic conditions (pH = 6.2) and air conditions. Figure 14c,d presents the MB decay in photocatalytic and control processes. The changes in the concentration of methylene blue (MB) over time in reaction with samples TaCuN-a, TaCuN-b, and TaCuN-c do not correspond to any kinetics of the degradation reaction. The observed concentration fluctuation during the degradation process may be caused by the adsorption–desorption equilibrium of MB on the material surface. The observed decay of MB is involved rather with photolysis than photocatalysis.

The catalytic degradation of MB on the tested intermetallic surfaces was negligible, likely due to both the high reflectance of the layers and the weak adsorption of MB on the surfaces. Adsorption of organic molecules occurs best on porous materials with a large specific surface area and a surface charge. Adsorption of an organic molecule on the catalyst surface is necessary for heterogeneous catalysis to occur. Contact between the substrate and the catalytically active site on the catalyst surface is a key element in enabling the reaction to proceed.

Scientific literature confirms that crystalline Ta_3_N_5_ nanostructures with high surface area demonstrate excellent visible-light photocatalytic activity for MB degradation [32]. Similarly, recent studies of Xiao et al. [33] revealed that Ta_3_N_5_ had two absorption edges at around 590 nm (~2.1 eV) and 480 nm (~2.6 eV), which are attributed to photon absorption. This optical anisotropy leads to less efficient light absorption in the wavelength range of 480–590 nm in Ta_3_N_5_ thin films. The addition of copper to tantalum nitride (forming TaCu) can modify its properties, but there is a lack of information on CuTaN layer photocatalytic activity. Studies on TaCu coatings indicate that varying copper content affects the material’s characteristics, including its antibacterial properties and possibly its optical absorption [20].

The metallic conduction path increased electron density but simultaneously reduced photon absorption and surface charge separation—key parameters for generating reactive species in photocatalysis. Furthermore, the small surface area and smooth morphology limited dye adsorption, meaning that methylene blue degradation occurred primarily through photolysis rather than heterogeneous photocatalysis.

### 3.4. Antibacterial Tests

**Figure 15:**
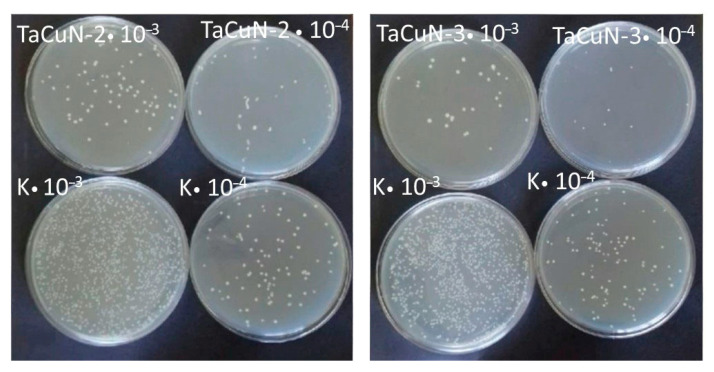
Grown colonies after 4 h incubation of bacteria on the tested coatings. Visible inhibition of growth on TaCuN-b and TaCuN-c in relation to K-control (glass plate).

The antimicrobial properties of samples TaCuN-b and TaCuN-c were assessed by measuring the percentage reduction in bacterial growth (%R) (Figure 15). The experiment was performed in duplicate, performing five and six independent experiments (Table 5) for TaCuN-b and TaCuN-c samples, respectively.

The mean reduction values for the tested samples were: 46.3 ± 19.6 and 77.5 ± 16.4 for sample TaCuN-b and TaCuN-c, respectively. The obtained results demonstrate higher antimicrobial efficacy of sample TaCuN-c compared to sample TaCuN-b. In the first trial, a result of 95.6% was achieved, which is close to the value for strong antibacterial materials such as TiO_2_/Ag or ZnO [34]. A large variability in efficacy was observed for sample TaCuN-b, with the minimum value being only 18.7%. The higher activity of TaCuN-c may result from the effective generation of reactive oxygen species by this sample.

One of the most important factors affecting the ability of bacteria to colonize biomaterial surfaces is their smoothness, which is expressed as surface roughness (*R*_a_). Increased surface roughness creates favorable conditions for bacterial adhesion [35]. This occurs due to the increased contact surface and the presence of microcracks and cavities where bacteria can settle and undergo cell division, resulting in the formation of a biofilm on the colonized surface. In the case of biomaterials such as titanium and its alloys, surface properties play a crucial role in the process of osseointegration and infection prevention [35]. Numerous studies have confirmed that surfaces characterized by high roughness (*R*_a_ = 0.5–1 μm) favor the adhesion of bacteria such as Staphylococcus aureus and Pseudomonas aeruginosa. Studies [35] confirm that increasing the surface roughness of titanium results in the formation of more bacterial biofilm. Surfaces characterized by a high degree of smoothness (*R*_a_ < 0.2 μm) present low susceptibility to bacterial colonization. This limitation stems from the inability of microorganisms to mechanically anchor, which prevents the formation of a stable biofilm structure. Barbour and colleagues [36] demonstrated that polished surfaces characterized by low roughness are resistant to bacterial colonization. *R*_a_ and *S*_a_ (measured in present work) describe the same physical concept—surface roughness—but in different measurement dimensions. *R*_a_ is the arithmetic average deviation of height values measured along a single 2D line profile. Sa is the areal (3D) analogue of *R*_a_. *S*_a_ is the arithmetic mean height deviation calculated over a 3D surface area. AFM measurements show that TaCuN-b has the highest nanoscale surface roughness in the series (*S*_a_ = 9.1 nm), whereas TaCuN-c is the smoothest (*S*_a_ = 3.4 nm). Increased roughness is known to promote heterogeneous bacterial adhesion, which naturally increases variability in %R outcomes.

Another factor influencing % R variability may be chemical composition of surfaces. TEM/EDS analysis demonstrates that the films possess a multilayered, compositionally non-uniform structure. In samples with intermediate Cu content, such as TaCuN-b, this may lead to spatial variability in Cu-rich regions and consequently fluctuations in ROS generation efficiency, further contributing to the spread in antibacterial activity.

Increased electrical conductivity positively correlated with antibacterial efficacy, particularly in Cu-rich TaCuN-c films. The metallic zones enabled efficient electron transfer from Cu sites to surface oxygen and water molecules, promoting the formation of reactive oxygen species (ROS) such as superoxide and hydroxyl radicals. These ROS disrupted bacterial membranes, explaining the observed reduction of *Staphylococcus aureus* by up to 95.6%. Furthermore, high in-plane conductivity stabilized local electronic pathways essential for Cu^+^/Cu^2+^ redox cycling, inhibiting bacterial growth under visible light. Similar observations are described in the literature. Azamatov et al. showed that the antibacterial efficacy of TaCu coatings is influenced by the copper content and annealing temperature [20]. They showed TaCu coatings with approximately 10 wt% copper annealed at 600 °C demonstrated that the antibacterial effectiveness of the TaCu thin films was more pronounced against Gram-negative bacteria (*E. coli* and *P. aeruginosa*) than Gram-positive bacteria (*S. aureus* and *S. enterica*), which was probably associated with the thinner peptidoglycan layer of Gram-negative bacteria. Due to this fact, copper ions and reactive oxygen species (ROS) can more easily penetrate the cell, leading to a high flow of both copper ions and ROS into the cell and bacterial death. Similarly, Elangovan et al., in their work [6], compared TaCu coatings with a tantalum nitride/copper (TaN/Cu) nanocomposite coatings. TaN/Cu coatings with 10.46 at.% Cu showed significantly higher antibacterial activity against *Pseudomonas aeruginosa* than TaN.

## 4. Conclusions

This study demonstrates that carefully adjusting the copper content in tantalum nitride coatings allows the creation of multifunctional surfaces with tailored optical and antibacterial properties.

In conclusion, it can be said that the process of film deposition on magnetron with different power densities on metal targets leads to the formation of a multilayer structure with different ratios of elements such as Ta, Cu, N, C, and O. Four layers are formed. All these changes affect the antibacterial properties and electrical conductivity of the film, making them promising for future use.

In a single deposition cycle, layers were deposited on both a dielectric substrate and a metallic substrate. Electrical measurements of the layer on the dielectric substrate were performed with current flowing parallel to the layer surface. On the metallic substrate, measurements were taken with current flowing perpendicular to the layer surface. It was determined that conductivity anisotropy occurs, meaning that the conductivity along the layer is about 10^7^ times higher than in the measurements perpendicular to the layer surface. The higher conductivity measured along the layer is caused by the formation of a conductive channel inside the layer, parallel to the surface. This channel likely formed during deposition, probably due to nitrogen diffusion into the layer adjacent to the substrate and into the near-surface layer.

Increased conductivity limits photocatalytic performance through increased optical reflectance and reduced surface adsorption; conversely, it enhances antibacterial efficacy through enhanced electron transfer and ROS generation. These findings highlight the dual functional trade-off inherent in nitride-based conductive coatings—favoring antimicrobial activity over photocatalytic activity.

Reactive magnetron sputtering enabled formation of TaCuN multilayers with distinct compositional regions. The coatings’ combination of high in-plane conductivity and strong antibacterial activity, particularly at higher Cu contents, highlights their promise for biomedical and functional coating applications, though their photocatalytic efficiency remains limited.

## Figures and Tables

**Table 1:** Deposition parameters.

Parameters	TaCuN-a	TaCuN-b	TaCuN-c
Discharge voltage, V	Cu = 286. Ta = 302	Cu = 390. Ta = 300	Cu = 272. Ta = 306
Discharge current, A	Cu = 0.6. Ta = 3.00	Cu = 1.0. Ta = 3.0	Cu = 1.4. Ta = 3.0
Power, kW	Cu = 0.26. Ta = 1.78	Cu = 0.44. Ta = 1.80	Cu = 0.72. Ta = 1.86
Ar, sccm	75	75	75
N_2_, sccm	50	50	50
Base pressure, mbar	2 × 10^−5^	2 × 10^−5^	2 × 10^−5^
Working pressure, mbar	1.7 × 10^−3^	1.7 × 10^−3^	1.7 × 10^−3^

**Table 2:** Roughness of the tested coatings measured on the 2D surface (Figure 6a–c).

	Roughness Parameters of Tested Coatings		
	TaCuN-a	TaCuN-b	TaCuN-c
*S*_a_, nm	7.8	9.1	3.4
*S*_q_, nm	13.2	11.6	5.5
*S*_p_, nm	107.1	48.9	63.7
*S*_v_, nm	−44.4	−38.1	−16.9

*S*_a_—arithmetic mean deviation of the surface roughness height from the reference plane. *S*_q_—mean square deviation of the surface roughness height from the reference plane. *S*_p_—the highest surface roughness profile height. *S*_v_—the highest surface roughness profile depth.

**Table 3:** Elemental composition, expressed in atomic percent, in the regions numbered in Figure 7.

	Ti, %at.	Ta, %at.	Cu, %at.	N, %at.	C,%at.	O, %at.
Layer 1	77	8	-	8	-	5
Layer 2	6	29	40	12	8	3
Layer 3	3	42	37	-	13	2
Layer 4	2	29	42	12	10	3

**Table 4:** The overall TaCuN film composition (integrated) for the three deposition modes, obtained from EDS analysis.

Elements	O, %at.	C, %at.	N, %at.	Ta, %at.	Cu, %at.	Ti (Sublayer), %at.
TaCuN-a	5.2	7.45	7.8	26.45	28.6	24.5
TaCuN-b	3.4	7.8	8.0	27.15	30.2	23.45
TaCuN-c	2.7	5.6	11.3	28.7	32.35	19.35

**Table 5:** Antibacterial activity TaCuN-b and TaCuN-c shown as percentage of growth reduction (%R).

Exp. No	TaCuN-b (%R)	TaCuN-c (%R)
1	67.7	95.6
2	56.3	87.5
3	18.7	90.1
4	54.7	72.7
5	34.2	66.0
6		53.0
Average %R	46.3 ± 19.6	77.5 ± 16.4

## Data Availability

The original contributions presented in this study are included in the article. Further inquiries can be directed to the corresponding authors.
